# Parameter study of the high temperature MOCVD numerical model for AlN growth using orthogonal test design

**DOI:** 10.1038/s41598-021-87554-8

**Published:** 2021-04-23

**Authors:** Jiadai An, Xianying Dai, Lansheng Feng, Jieming Zheng

**Affiliations:** 1 School of Microelectronics, Xidian University, Xi’an, 710071 China; 2 State Key Discipline Laboratory of Wide Bandgap Semiconductor Technologies, Xidian University, Xi’an, 710071 China; 3 School of Mechano-Electronic Engineering, Xi’an, 710071 China

**Keywords:** Engineering, Applied physics, Fluid dynamics

## Abstract

We investigated the process parameters of the high temperature MOCVD (HT-MOCVD) numerical model for the AlN growth based on CFD simulation using orthogonal test design. It is believed that high temperature growth condition is favorable for improving efficiency and crystallization quality for AlN film, while the flow field in the HT-MOCVD reactor is closely related to the process parameters, which will affect the uniformity of the film. An independently developed conceptual HT-MOCVD reactor was established for the AlN growth to carry out the CFD simulation. To evaluate the role of the parameters systematically and efficiently on the growth uniformity, the process parameters based on CFD simulation were analyzed using orthogonal test design. The advantages of the range, matrix and variance methods were considered and the results were analyzed comprehensively and the optimal process parameters were obtained as follows, susceptor rotational speed 400 rpm, operating pressure 40 Torr, gas flow rate 50 slm, substrate temperature 1550 K.

## Introduction

As a wide range of direct bandgap semiconductor material, aluminum nitride (AlN) has been extensively investigated due to its high piezoelectric coefficient and thermal conductivity, and excellent optical and mechanical properties etc^1–4^, which make it a promising candidate for bulk substrate, blue and ultraviolet photo-detectors and deep ultraviolet LED^5–9^. Generally, metal organic chemical vapor deposition (MOCVD) is a typical and effective approach to the AlN film growth with TMAl/NH_3_ as the gas precursor^10–15^. At high growth temperature, AlN film can achieved high surface growth rate, and the resultant AlN crystal surface is atomically flat^16^. In recent years, some progress of high-quality LED research has been made in experiment, and fluid flow, heat transfer, mass transfer and chemical reactions during the AlN growth have received extensive attention^17,18^. The effects and mechanisms of varied temperature and pressure on chemical reaction pathways and rate were studied based on Grove theory^19^. The effects of flow rate on AlN growth rate was investigated by CFD simulation which found that the film growth rate increased with the increase of hydrogen flow rate^20^.

Traditional test methods would need a large number of experiments and analyses to obtain effective parametric research. Orthogonal test design is a multi-factor and multi-level multi-factor experimental research method, which is widely used to solve engineering application problems based on mathematical analysis^21,22^. In this paper, the optimal numerical simulation results can be obtained by the comprehensive optimization design of various parameters of multi-physical fields, a large amount of calculation data will be generated. The test points selected during the orthogonal test design are evenly distributed and uniformly comparable, which have strong representativeness and will meet the demand of comprehensive numerical simulation^23,24^. This study using an orthogonal test design to find out the best process conditions, the best test results, and gives the scheme of process optimization.

In this paper, the multi-physics fields and process parameters of AlN film growth by HT-MOCVD have been investigated by orthogonal test design. According to the factors and levels, an L_16_(4^4^) orthogonal array is employed to quantify the multi-physics fields and process parameters. The influence of process parameters for the optimal film uniformity was analyzed by range, matrix and variance analysis, respectively.

## Computation model and methods

### Geometry description

**Figure 1:**
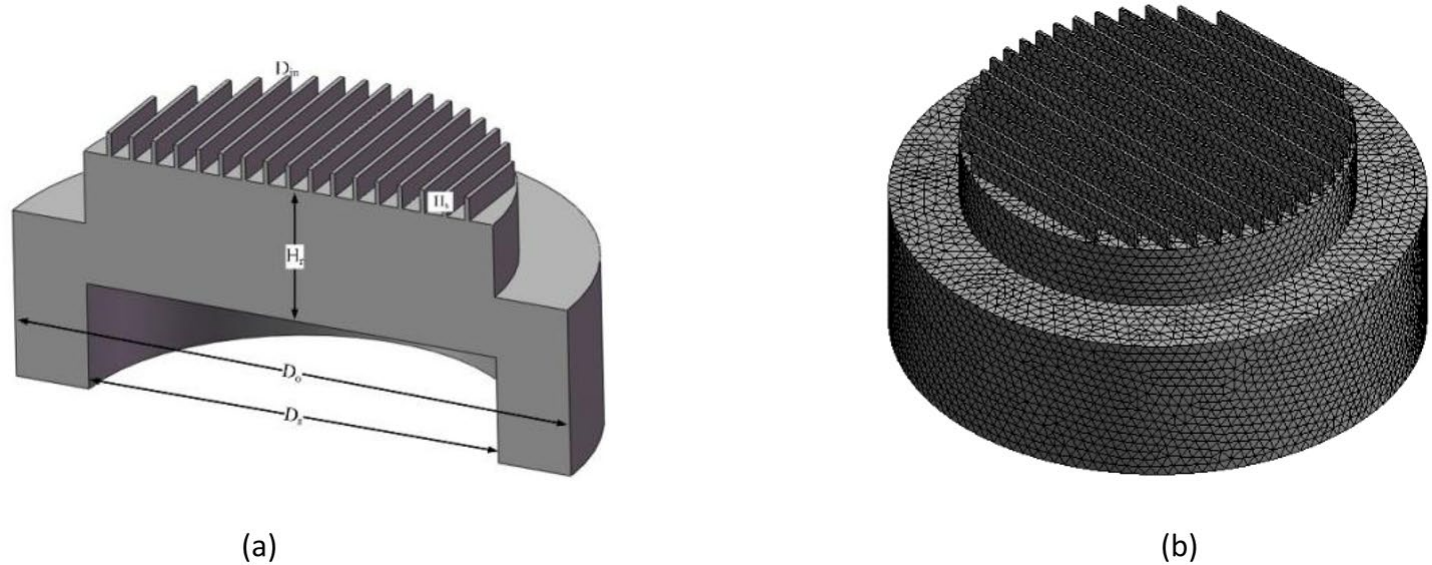
Model and mesh diagram of AlN-HT-MOCVD reactor. (**a**) Reactor model of the MOCVD reactor; (**b**) the whole mesh of AlN-MOCVD reactor.

**Figure 2:**
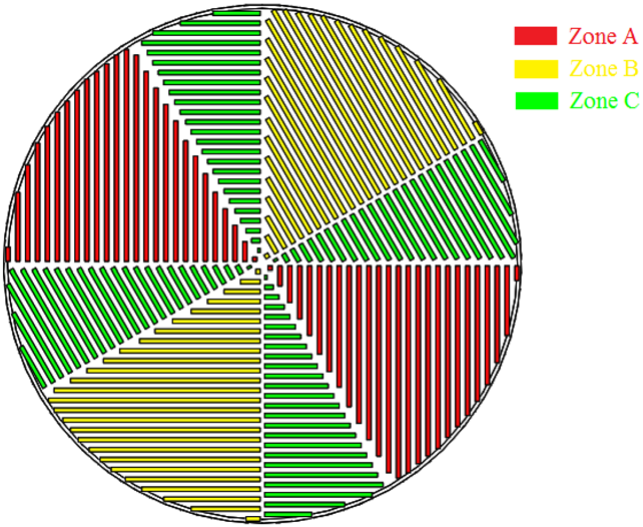
Diagram of isolation device.

Schematic diagram of a specific MOCVD reactor similar to the close-coupled showerhead (CCS) reactor is shown in Fig. 1a. The reactor consisted of the gas inlets, the chamber walls, the susceptor and the gas outlets. The gas inlets are used to transfer the group III metal organic precursors and group V gas hydrides carried by carrier gas (H_2_) into the reactor and the precursors gases pass through the chamber to participate in chemical reactions. The whole numerical simulation process is based on a three-dimensional model, which is divided into about one million grids as shown in Fig. 1b. The dimensions of the reactor are described in Fig. 1a which the height (H_s_), the width of gas inlet (D_in_), the susceptor diameter (D_s_), the outer wall diameter (D_o_) and the reactor height (H_r_) are 8 mm, 2 mm, 183 mm, 256 mm and 57.8 mm, respectively. As the reaction chamber is a vertical spray structure. Mixing of gaseous reactants into the reaction chamber may cause the parasitic reactions and produce more complex pre-reactions. Finally, it will affect the uniformity of film deposition. In order to prevent the metallic organic gas source and ammonia from mixing immediately after entering the inlets, we used a zoned isolation device at the inlets. Isolation is done by using carrier gas, which is N_2_ and H_2_ the inert gas. After the gas source enters the reaction chamber, it will spread in a vertical direction alone under the action of the isolation gas until it is close to the high temperature area of the substrate and then fully mix and react. To a certain extent, the occurrence of parasitic reactions and partial waste of gas sources are avoided. The partition isolation device is shown in Fig. 2, in which area A is TMAl, area B is NH_3_, and area C is N_2_ and H_2_.

### Orthogonal test design

The orthogonal test design that optimizing the multi-physics fields and process parameters of the AlN growth by HT-MOCVD was divided into several parts: selecting test objectives, evaluating indicators, selecting factors and levels, designing an appropriate orthogonal array, listing test plan and corresponding results, analyzing the test results through range, matrix and variance analysis, and finally finding the optimal combination of factors and levels.

During AlN growth by HT-MOCVD the reactants are TMAl, NH_3_, hydrogen and other gases, and the hydrodynamic behavior and flow field distribution in the reactor will affect the uniformity of the film. The hydrodynamic behavior is closely related to the (A) gas flow rate, (B) operating pressure, (C) substrate temperature, and (D) rotating speed of susceptor. As the test involves 4 degrees of freedom and the complex interaction among them, a large number of experiments are needed to find out the most stable flow field distribution and relatively optimal combination of process parameters.

In the design of orthogonal test, the core issue is to select and identify the factors that may affect the indicators. Therefore, taking no account of the interaction influence among these parameters, the factors can be considered as the variables which correspond to the four free parameters i.e. A, B, C, and D mentioned above. Level, also known as bit-level, corresponding to the factors represented different numerical simulation states. We should consider both the range of parameters and the number of levels to determine the level, so the factors were divided into four continuous levels to represent the variation range. Flow field and heat transfer in the reactor need to be considered and the continuous liquid level should be appropriated. The orthogonal factors and levels are shown in Table 1.

**Table 1:** Orthogonal factor and level table.

Factors	Level				Unit
	1	2	3	4	
A Total gas flow rate	45	50	55	60	slm
B Operating pressure	40	50	60	70	Torr
C Substrate temperature	1450	1500	1550	1600	K
D Rotating speed	400	600	800	1000	rpm

**Figure 3:**
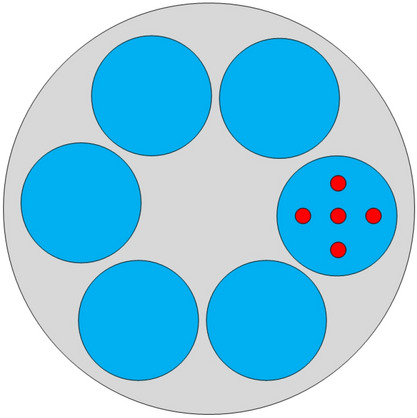
5-point positions schematic diagram.

In this study, we have investigated four free parameters and each including four levels. The L_16_(4^4^) orthogonal array was adopted to carry out the optimization combination test. Where L refers to orthogonal array, the 16 represents that the main array has sixteen rows, which indicates 16 cases designed by orthogonal array, the 4 represents that each investigated factors has four levels, and the 5 denotes that the test has five columns, which indicates five factors. As shown in Fig. 3, in order to analyze the influence of the four factors on the three indicators, 5 points on the substrate were selected according to the 5 point principle of wafer uniformity. The variance of the simulation results is calculated as

1 $$ S^{2} { = }{{\sum\limits_{1}^{{\text{n}}} {(x_{i} - m)^{2} } } \mathord{\left/ {\vphantom {{\sum\limits_{1}^{{\text{n}}} {(x_{i} - m)^{2} } } {(n - 1)}}} \right. \kern-\nulldelimiterspace} {(n - 1)}} $$

where *x*_*i*_ is the data of one point, *m* is the average value of *x*_*i*_, and *n* is the number of data.

**Figure 4:**
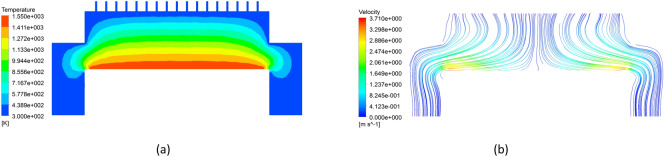
Distributions of fields (**a**) temperature and (**b**) velocity magnitude in the reactor.

**Figure 5:**
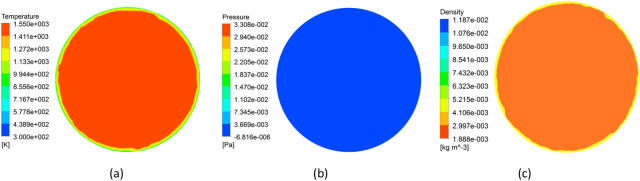
Distributions of fields on the substrate. (**a**) temperature (**b**) pressure (**c**) density.

As shown in Table 2, the first column lists the numbers from 1 to 16, representing the 16 simulation cases. Columns 2 to 5 represent levels of different factors, i.e. A, B, C and D. Considering that the numerical simulation in this study involves four changing factors, the last column in the table is error evaluation, namely error term. In Table 1, each row in the table corresponds to a growth condition, where the numbers “1, 2, 3, 4” represent the different levels of each factor. For example, the 5th case corresponds to a combination of 2nd level of A, the 1st level of B, the 2ed level of C and the 3rd level of D. The right columns on the right side of the table list the indicators, namely the density variance, pressure variance, and temperature variance for each case. During this study the sixteen simulation cases were investigated using CFD numerical method and the results of the 14th case are shown in Figs. 4 and 5.

**Table 2:** L_16_(4^4^) orthogonal designed scheme and results.

Case	A	B	C	D	Error term	Density variance	Pressure variance	Temperature variance
1	1	1	1	1	1	4.4211 × 10^−11^	6.0372 × 10^−8^	1.3966 × 10
2	1	2	2	2	2	1.3856 × 10^−10^	3.3669 × 10^−7^	1.1943 × 10
3	1	3	3	3	3	3.9001 × 10^−10^	9.0930 × 10^−7^	1.0605 × 10
4	1	4	4	4	4	4.2459 × 10^−10^	1.6954 × 10^−6^	1.5956 × 10
5	2	1	2	3	4	8.0444 × 10^−11^	6.4854 × 10^−7^	1.1331 × 10
6	2	2	1	4	3	2.6294 × 10^−10^	1.4698 × 10^−6^	0.9650 × 10
7	2	3	4	1	2	1.1161 × 10^−10^	8.7931 × 10^−8^	1.6038 × 10
8	2	4	3	2	1	2.0482 × 10^−10^	1.0645 × 10^−7^	1.3949 × 10
9	3	1	3	4	2	9.3821 × 10^−11^	1.2373 × 10^−6^	1.1044 × 10
10	3	2	1	3	1	1.9523 × 10^−10^	7.2691 × 10^−7^	0.9417 × 10
11	3	3	4	2	4	2.1107 × 10^−10^	3.2333 × 10^−7^	1.3227 × 10
12	3	4	2	1	3	2.0932 × 10^−10^	1.0082 × 10^−7^	1.2661 × 10
13	4	1	4	2	3	5.0425 × 10^−11^	2.0195 × 10^−7^	1.5945 × 10
14	4	2	3	13	4	6.7071 × 10^−11^	5.8788 × 10^−8^	1.5671 × 10
15	4	3	2	4	1	4.5579 × 10^−10^	1.3709 × 10^−6^	1.2380 × 10
16	4	4	1	3	2	4.8599 × 10^−10^	6.8550 × 10^−7^	1.1808 × 10

According to the design above, for any column, all four levels were participated and occur with the same frequency. Two columns contain any possible combination of levels and the occurrences are equal. Each layer of a factor is equal to the combination of factors of the other layers, indicating that the horizontal configuration between any two columns is consistent.

## Results and discussion

### Range analysis

Judging the order and optimal level of each factor by the average value of each factor based on the orthogonal test design. The judgment methods are as follows: (1) K_1_, K_2_, K_3_, K_4_ represent the average value of each factor at each level. By comparing the mean of the levels to determine the factors of the optimal level, the smaller the $\overline{{K_{{\text{i}}} }}$, the smaller the variance of the experimental results, the better the uniformity caused by the case. (2) Range R = max {K_1_, K_2_, K_3_, K_4_} − min {K_1_, K_2_, K_3_, K_4_} indicates the influence of various factors. The large range indicates that the horizontal variation of this factor has a great influence on the experimental result which is the main factor. The range of error term indicates the error caused by the random. If the range of error term is large, an interaction between various factors exits. Through the simulation of 16 orthogonal test cases, the order of each factor and the optimal level of density distribution, pressure distribution and temperature distribution were obtained. As the Table 3 shows, the range of error term in the three indicators is smaller than that of other factors, indicating that the interaction among factors is weak. Therefore, the orthogonal test design is reasonable and there is no need for interactive design.

**Table 3:** Optimal combination of process parameters with single index.

Index	Indicator	Factor				
		A	B	C	D	Error term
Density distribution	$\overline{K}_{1}$	2.4934 × 10^−10^	6.7225 × 10^−11^	2.4709 × 10^−10^	1.0805 × 10^−10^	2.2501 × 10^−10^
	$\overline{K}_{2}$	1.6495 × 10^−10^	1.6595 × 10^−10^	2.2103 × 10^−10^	1.5122 × 10^−10^	1.9994 × 10^−10^
	$\overline{K}_{3}$	1.7736 × 10^−10^	2.9212 × 10^−10^	1.8893 × 10^−10^	2.8792 × 10^−10^	2.2817 × 10^−10^
	$\overline{K}_{4}$	2.6482 × 10^−10^	3.3118 × 10^−10^	3.0832 × 10^−10^	3.0928 × 10^−10^	1.9579 × 10^−10^
	R	9.9865 × 10^−11^	2.6395 × 10^−10^	1.1939 × 10^−10^	2.0123 × 10^−10^	3.2380 × 10^−11^
	Order	B_1_–D_1_–C_3_–A_2_				
Pressure distribution	$\overline{K}_{1}$	7.5043 × 10^−7^	5.3705 × 10^−7^	7.3565 × 10^−7^	7.6979 × 10^−8^	5.6615 × 10^−7^
	$\overline{K}_{2}$	5.7819 × 10^−7^	6.4805 × 10^−7^	6.1423 × 10^−7^	2.4210 × 10^−7^	5.8686 × 10^−7^
	$\overline{K}_{3}$	5.9710 × 10^−7^	6.7286 × 10^−7^	5.7797 × 10^−7^	7.4256 × 10^−7^	6.7048 × 10^−7^
	$\overline{K}_{4}$	5.7927 × 10^−7^	6.4703 × 10^−7^	5.7714 × 10^−7^	1.4433 × 10^−6^	6.8150 × 10^−7^
	R	1.7224 × 10^−7^	1.3581 × 10^−7^	1.5851 × 10^−7^	1.3664 × 10^−6^	1.1535 × 10^−7^
	Order	D_1_–A_2_–C_4_–B_1_				
Temperature distribution	$\overline{K}_{1}$	13.1174	13.0717	11.2102	14.5840	12.4282
	$\overline{K}_{2}$	12.7422	11.6702	12.0788	13.7660	12.7081
	$\overline{K}_{3}$	11.5874	13.0626	12.8173	10.7903	12.2153
	$\overline{K}_{4}$	13.9510	13.5935	15.2916	12.2576	14.0463
	R	2.3637	1.9232	4.0814	3.7937	1.8310
	Order	C_1_–D_3_–A_3_–B_2_				

As the range of B (2.6395 × 10^–10^) > the range of D (2.0123 × 10^–10^) > the range of C (1.1939 × 10^–10^) > the range of A (9.9865 × 10^–11^), which affects the density distribution is B, D, C, A. Therefore, the optimal level combination of density distribution is B_1_–D_1_–C_3_–A_2_. Similarly, the optimal level combination of pressure distribution and temperature distribution is D_1_–A_2_–C_4_–B_1_ and C_1_–D_3_–A_3_–B_2_.

### Matrix analysis

The order of the factors and the optimal level were judged by the range analysis, while the weight of each factor and level should be analyzed to determine the optimal combination using the matrix analysis. As the index layer, factor layer and level layer are the three-layer structural models of the orthogonal test, we defined 1, index layer matrix: including m factors, each factor is divided into n levels, and the average index of the j level of factor A_i_ is *K*_*ij*_. The surface of the film is expected to be uniform, and the matrix was

2 $$ M = \left( {\begin{array}{*{20}c} {K_{11} } & 0 & \cdots & 0 \\ {K_{12} } & 0 & \cdots & 0 \\ \vdots & \vdots & \ddots & \vdots \\ 0 & 0 & 0 & {K_{{{\text{mn}}}} } \\ \end{array} } \right). $$

Definition 2, factor layer matrix, $T_{{\text{i}}} { = }{1 \mathord{\left/ {\vphantom {1 {\sum\nolimits_{j = 1}^{{\text{n}}} {K_{{{\text{ij}}}} } }}} \right. \kern-\nulldelimiterspace} {\sum\nolimits_{j = 1}^{{\text{n}}} {K_{{{\text{ij}}}} } }}$, matrix T was established as

3 $$ T = \left( {\begin{array}{*{20}c} {T_{1} } & 0 & \cdots & 0 \\ 0 & {T_{2} } & \cdots & 0 \\ \vdots & \vdots & \ddots & \vdots \\ 0 & 0 & 0 & {T_{{\text{m}}} } \\ \end{array} } \right). $$

Definition 3, level layer matrix, the range of A_1_ is s_i_, $S_{{\text{i}}} { = }{{{\text{s}}_{i} } \mathord{\left/ {\vphantom {{{\text{s}}_{i} } {\sum\nolimits_{i = 1}^{m} {s_{i} } }}} \right. \kern-\nulldelimiterspace} {\sum\nolimits_{i = 1}^{m} {s_{i} } }}$, matrix S was established as

4 $$ S^{T} { = }\left( {\begin{array}{*{20}c} {S_{1} } & {S_{2} } & \cdots & {S_{m} } \\ \end{array} } \right) $$

The range analysis method of multi-index orthogonal experiment is summarized.

*Step 1* Find the K matrix, T matrix and S matrix of each factor, and then calculate the weight matrix *k*_*i*_ (i = 1, …, m).

Step 2: Take the mean matrix of the m matrices ${{\sum\nolimits_{{{\text{i}} = 1}}^{{\text{m}}} {{\text{k}}_{{\text{i}}} } } \mathord{\left/ {\vphantom {{\sum\nolimits_{{{\text{i}} = 1}}^{{\text{m}}} {{\text{k}}_{{\text{i}}} } } {\text{m}}}} \right. \kern-\nulldelimiterspace} {\text{m}}}$ as follows

5 $$ {\text{k}}^{T} { = }\left( {\begin{array}{*{20}c} {A_{11} } & \cdots & {A_{{1{\text{n}}}} } & \cdots & {A_{{{\text{m}}1}} } & \cdots & {A_{{{\text{mn}}}} } \\ \end{array} } \right) $$

where $A_{ij} \left( {i = 1 \cdots M,j = 1 \cdots N} \right)$ indicator values corresponding to the jth level of the first factor.

*Step 3* According to the requirements of the test for each index, find out the level j corresponding to the maximum or minimum value of each A_i_ in the K matrix, regard it as the optimal level of the corresponding factors, and then find out the optimal level combination of all factors.

According to the above calculation steps, the weight matrix of the temperature distribution is calculated as follows

6 $$ T_{3} = \left( {\begin{array}{*{20}c} {0.313} & 0 & 0 & 0 \\ 0 & {0.312} & 0 & 0 \\ 0 & 0 & {0.315} & 0 \\ 0 & 0 & 0 & {0.315} \\ \end{array} } \right) $$

7 $$ S_{3} = \left( {\begin{array}{*{20}c} {0.194} \\ {0.158} \\ {0.336} \\ {0.312} \\ \end{array} } \right) $$

8 $$ M_{3} = \left( {\begin{array}{*{20}c} {0.076} & 0 & 0 & 0 \\ {0.078} & 0 & 0 & 0 \\ {0.086} & 0 & 0 & 0 \\ {0.072} & 0 & 0 & 0 \\ 0 & {0.077} & 0 & 0 \\ 0 & {0.086} & 0 & 0 \\ 0 & {0.077} & 0 & 0 \\ 0 & {0.074} & 0 & 0 \\ 0 & 0 & {0.089} & 0 \\ 0 & 0 & {0.083} & 0 \\ 0 & 0 & {0.078} & 0 \\ 0 & 0 & {0.065} & 0 \\ 0 & 0 & 0 & {0.069} \\ 0 & 0 & 0 & {0.073} \\ 0 & 0 & 0 & {0.093} \\ 0 & 0 & 0 & {0.082} \\ \end{array} } \right) $$

9 $$ k_{3} = M_{3} T_{3} S_{3} = \left( {\begin{array}{*{20}c} {0.0046} \\ {0.0048} \\ {0.0052} \\ {0.0044} \\ {0.0038} \\ {0.0042} \\ {0.0038} \\ {0.0036} \\ {0.0094} \\ {0.0088} \\ {0.0083} \\ {0.0069} \\ {0.0067} \\ {0.0071} \\ {0.0091} \\ {0.0080} \\ \end{array} } \right) $$

10 $$ k_{1} = \left( {\begin{array}{*{20}c} {0.1141} \\ {0.1723} \\ {0.1604} \\ {0.1074} \\ {1.5688} \\ {0.6356} \\ {0.3610} \\ {0.3185} \\ {0.1207} \\ {0.1350} \\ {0.1580} \\ {0.0968} \\ {0.6142} \\ {0.4390} \\ {0.2305} \\ {0.2146} \\ \end{array} } \right). $$

Similarly, the weight matrix K_2_ of the density distribution and the weight matrix K_3_ of the pressure distribution are calculated. Finally, the weight matrix which affects the index is obtained

11 $$ k_{2} = \left( {\begin{array}{*{20}c} {0.0081} \\ {0.0105} \\ {0.0102} \\ {0.0105} \\ {0.0089} \\ {0.0074} \\ {0.0071} \\ {0.0074} \\ {0.0076} \\ {0.0091} \\ {0.0097} \\ {0.0097} \\ {1.8555} \\ {0.5900} \\ {0.1923} \\ {0.0990} \\ \end{array} } \right) $$

12 $$ k = \frac{{k_{1} + k_{2} + k_{3} }}{3} = \left( {\begin{array}{*{20}c} {0.0422} \\ {0.0624} \\ {0.0585} \\ {0.0406} \\ {0.5272} \\ {0.2159} \\ {0.1240} \\ {0.1100} \\ {0.0460} \\ {0.0510} \\ {0.0587} \\ {0.0379} \\ {0.8254} \\ {0.3454} \\ {0.1441} \\ {0.1073} \\ \end{array} } \right). $$

Based on the above calculation and analysis, the optimum combination is D_1_–B_1_–A_2_–C_3_. The optimum process parameters for the AlN growth by HT-MOCVD are as follows, the susceptor rotational speed 400 rpm, the operating pressure 40 Torr, the gas flow rate 50 slm, the substrate temperature 1550 K.

## Variance analysis

The matrix analysis method can obtain the optimum process parameters, while the cause of the difference of experiment results at different levels of factors is not clear. The analysis of variance can not only give an accurate estimate of the impact of each factor on the test results, but also provide a criterion to judge whether the effect of the factors examined is significant. We performed variance analysis and the results are shown in Table 4.

**Table 4:** Analysis of variance of orthogonal experiment.

Index	Factor	*S*	*f*	*V*	$F_{{\text{a}}}$	$F_{\theta }$	Significance level
Density distribution	A	1.8949 × 10^−21^	3	6.3165 × 10^−22^	9.04	F_0.10_(3,3) = 5.39	Significant
	B	1.0921 × 10^−20^	3	3.6405 × 10^−21^	52.09	F_0.05_(3,3) = 9.28	Significant
	C	1.9196 × 10^−21^	3	6.3986 × 10^−22^	9.16	F_0.025_(3,3) = 15.4	Insignificant
	D	7.4274 × 10^−21^	3	2.4758 × 10^−21^	35.43	F_0.005_(3,3) = 47.5	Insignificant
	e	2.0966 × 10^−22^	3	6.9886 × 10^−23^			
Pressure distribution	A	5.1967 × 10^−15^	3	1.7322 × 10^−15^	2.04	F_0.10_(3,3) = 5.39	Insignificant
	B	2..7591 × 10^−15^	3	9.1969 × 10^−16^	1.09	F_0.05_(3,3) = 9.28	Insignificant
	C	4.2141 × 10^−15^	3	1.4047 × 10^−15^	1.66	F_0.025_(3,3) = 15.4	Insignificant
	D	2.8261 × 10^−13^	3	9.4203 × 10^−14^	111.1	F_0.005_(3,3) = 47.5	Significant
	e	2.5430 × 10^−15^	3	8.4768 × 10^−16^			
Temperature distribution	A	0.7224	3	2.4080 × 10^−1^	1.42	F_0.10_(3,3) = 5.39	Insignificant
	B	0.5097	3	1.6991 × 10^−1^	1.00	F_0.05_(3,3) = 9.28	Insignificant
	C	2.3116	3	7.7052 × 10^−1^	4.55	F_0.025_(3,3) = 15.4	Insignificant
	D	2.1098	3	7.0325 × 10^−1^	4.15	F_0.005_(3,3) = 47.5	Insignificant
	e	0.5080	3	1.6934 × 10^−1^			

In the variance analysis of orthogonal test, the variance of error term is used as the estimation of test error. The formulas used in variance analysis are as follows

13 $$ S{ = }\sum\limits_{i = 1}^{K} {\frac{{T_{i}^{2} }}{K}} - \frac{{T^{2} }}{n} $$

14 $$ f = K - 1 $$

15 $$ V = \frac{S}{f} $$

16 $$ F_{\theta } = \frac{{V_{f} }}{{V_{\theta } }} $$

where *S* is the sum of deviation squares, *K* is the total number of factors, *n* is the total number of experiments, *T*_*i*_ is the sum of the data corresponding to level *i*, *T* is the sum of all experimental data, *f* is the degree of freedom of factors, *V* is the mean square, $F_{\theta }$ is the ratio of *F*, *V*_*f*_ is the mean square of factors, and *V*_*θ*_ is the mean square of errors.

According to the theory of mathematical statistics, if $F_{{\text{a}}}$ is greater than $F_{\theta }$, it is considered that this factor has a significant effect on the results; if $F_{{\text{a}}}$ is less than $F_{\theta }$, it is considered that this factor has no significant effect on the results. As shown in Table 4, for density distribution, according to $F_{\theta }$, the order of factors can be determined as B → D → C → A, in which the $F_{\theta }$ of pressure is much larger than *F*_0.05_ (3,3), and the pressure factor is very significant; The pressure distribution is determined by $F_{\theta }$ to determine the order of factors as D → A → C → B. The temperature distribution is determined by $F_{\theta }$ to determine the order of factors as C → D → A → B. Through the evaluation of variance method, the optimal parameters obtained were verified, and the criterion to judge whether the effect of the factors examined is significant can provide theoretical guidance for optimization of process parameters during HT-MOCVD growth.

## Conclusion

In this paper, an optimized multi-physical field numerical model was established for the AlN growth by high temperature MOCVD (HT-MOCVD) based on CFD simulation and the influences of the process parameters on the AlN growth uniformity were investigated carefully based on the orthogonal test design. Firstly, the orthogonal test design takes the susceptor rotational speed, operating pressure, gas flow rate, substrate temperature as factors, and the density field distribution, pressure field distribution, temperature field distribution as indicators. Then, the data of 5 points on the surface were extracted to quantify the film uniformity. Finally, the results were analyzed by range, matrix and variance methods considering the influence of process parameters on film uniformity. The optimum process parameters of this three-dimensional reactor for the AlN growth by HT-MOCVD were obtained as follows: the susceptor rotational speed 400 rpm, operating pressure 40 Torr, gas flow rate 50 slm, substrate temperature 1550 K.

## Data Availability

The datasets used and/or analysed during the current study are available from the corresponding author on reasonable request.

## Abbreviations

HT-MOCVD: High temperature metal organic chemical vapor deposition
CFD: Computational fluid dynamics
TMAl: Trimethylaluminium
